# Correction: Membranes, Energetics, and Evolution Across the Prokaryote-Eukaryote Divide

**DOI:** 10.7554/eLife.35006

**Published:** 2018-07-05

**Authors:** Michael Lynch, Georgi Marinov

Lynch M, Marinov GK. 2018. Membranes, energetics, and evolution across the prokaryote-eukaryote divide. *eLife* **6**:e20437. doi: 10.7554/eLife.20437.

Published 16, March 2017

A reader has pointed out that Appendix 1—table 3 of this paper was incomplete and also contained a number of errors. We have corrected the errors (see below) and have also corrected Figure 2, which was based on the data in this table. We have also added a source data file that includes all of the data used to plot Figure 2, including full references to the original sources for each data point: where there are multiple estimates per species, these have been averaged.

**Figure 2.**
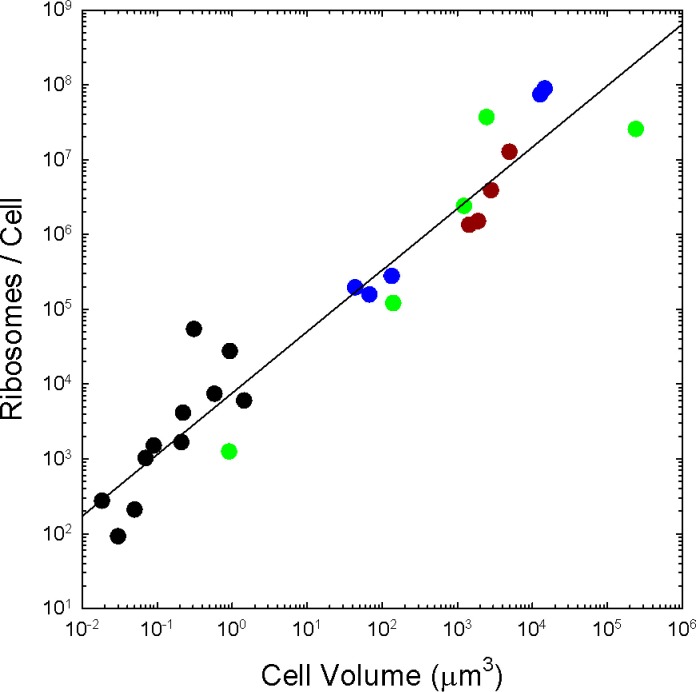
The number of ribosomes per cell scales with cell volume (V, in μm^3^) as 7586V^0.82^ (r^2^ = 0.92; SEs of the intercept and slope on the log scale are 0.13 and 0.05, respectively). Color coding as in previous figures. The data presented in this figure can be found in Figure 2—source data 1; see also Appendix 1—table 3. Figure 2—source data 1.

The cell volume data in Appendix 1—table 3 for the following species have been corrected: *B. subtilis*, *E. coli*, *S. alaskensis*, *S. aureus*, *S. ceres*, *S. pombe*, *T. pyriformis*, *T. thermophila*, *C. reinhardtii* and HeLa cell; some data are also quoted to fewer digits than before. The ribosome number data in the table for the following species have been corrected: *L. interrogans*, *M. pneumonii*, *S. ceres*, *S. pombe*, *T. pyriformis*, *A. aestivalis* and HeLa cell. The caption for this table has also been updated.

We have also replotted Figure 2 with the corrected data (see below) and the end result is very similar, but with a slightly improved regression relative to the previous version.

